# A crisis mode in migration governance: comparative and analytical insights

**DOI:** 10.1186/s40878-022-00284-2

**Published:** 2022-03-21

**Authors:** Zeynep Sahin-Mencutek, Soner Barthoma, N. Ela Gökalp-Aras, Anna Triandafyllidou

**Affiliations:** 1 Bonn International Center for Conflict Studies, BICC, Pfarrer-Byns-Straße 1, 53121 Bonn, Germany; 2 Ryerson University, CERC, Toronto, Canada; 3 Uppsala University, Engelska Parken, Thunbergsvägen 3C, 751 20 Uppsala, Sweden; 4 Swedish Research Institute in Istanbul (SRII), Istiklal Caddesi 247 P.K. 344 33, Tunel-Beyoglu, Istanbul, Turkey; 5 Department of Sociology, CERC Migration, Ryerson University, 350 Victoria Street, Toronto, 5MB 2K3 Canada

**Keywords:** Crisis, Asylum, Refugee emergency, Legal fragmentation, The multiplicity of actors, Renationalisation, Temporary protection

## Abstract

This paper takes stock of the emerging literature on the governance and framing of both migration and asylum as ‘crises’. This study carries forward this line of thinking by showing how the crisis governance of migration is not just a representation or a discourse but emerges as a mode of governance with specific features. The study focuses on the refugee emergency of 2015–2016, covering however a longer time frame (2011–2018) and a wide set of 11 countries (those neighbouring Syria: Lebanon, Iraq and Turkey; countries that were mainly transit points: Greece, Italy, Poland and Hungary; and countries that were mainly destination points (Austria, Germany, Sweden and the UK). Through the meta-analysis of a broad set of materials arising out of the RESPOND research project, we identified three interacting governance features in times of crisis. These include (1) a multilevel but complex actor landscape (2) complicated and fragmented legal systems and policy provisions that may vary both at the temporal and territorial level; (3) a renationalisation narrative that seeks to bring this multifaceted and fragmented governance landscape together under the promise that the national state can re-establish control and solve the ‘crisis.’

## Introduction

Migration is part and parcel of human history and an inherent feature of social and economic growth and transformation. And yet, during the last 30 years, migration, particularly when it happens outside regulated schemes and controlled pathways, has been primarily represented and conceptualized as a ‘crisis’, as an abnormal event that disrupts the ordinary course of social and economic activity (Martin et al., 2014). In Europe, framing migration as a crisis has reinforced a securitized view of migration, making it a top concern in the European Union (EU) policy agenda (focusing particularly on the management of forced migration arising from conflicts in Syria, Iraq, Afghanistan, Libya and elsewhere) (Estevens, 2018). Indeed 2015 was a ‘crisis year’ with over 1.8 million irregular border-crossings at the EU’s external borders (1,822,337 in 2015), of which nearly half (885,386) (Eurostat, 2017) were recorded at the Eastern Mediterranean route from Turkey to Greece. Following these flows, asylum applications reached their peak in 2015 at 1,257,000 (Eurostat, 2021), reinforcing the usage of the term ‘crisis’ to speak about both migration and asylum-seeking.

The concept of crisis is contested—as to crisis of what and for whom? Was 2015 a crisis for Syrians seeking refuge in other countries, or was it a crisis for transit and destination countries in Europe because they felt that migration and asylum flows were out of control? And what was in crisis, governance mechanisms, reception centres, welfare systems or border guard capacity? Menjívar et al. (2019: 2) rightly notes that “the term ‘crisis,’ is overused in today’s society, and its meaning is somehow diffused: today’s crises are becoming the “new norm”. In some definitions, the ‘asylum crisis’ or the ‘refugee crisis’ semantically addresses ‘refugees’ or ‘migrants’ as the main cause of the ‘crisis’, rather than investigating the real causes of the emergency (Crawley & Skleparis, 2018). In some other definitions, the notion of crisis is used as a signifier to explain its consequences on different actors and systems, notably on “Europe”, the “EU” or “the EU Member States”, such as the “European refugee crisis”, the “European humanitarian crisis” (Carrera et al., 2019; Dines et al., 2018), the European “solidarity crisis” (Grimmel & Giang, 2017), “Europe in crisis”, and as an identity and a “racial crisis that derives fundamentally from the postcolonial condition of “Europe” as a whole” (De Genova, 2018: 1765).

A crisis is commonly identified as an extraordinary event leading to increased but temporal instability and uncertainty in the pre-existing status quo or perceived ‘normality’. Policy and governance studies have been long interested in understanding policy processes in the context of financial, humanitarian, energy, and health crisis at national and international levels. Their first observation is that as there is no objective measure for identifying a ‘disruptive event’ as a crisis, the events need to be perceived as a crisis (Grossman, 2019). Policy makers and implementers try to make sense of the highly dynamic context during a ‘crisis’ through processes of naming, selecting and storytelling (Rein & Schön, 1996). A crisis often calls forth public policy responses, but the events themselves are often clouded by uncertainty and ambiguity (Grossman, 2019). Through cognitive framing, governance actors develop these policy responses (Geddes & Abd-Houj, 2018).

A second point arising from the governance literature is that diverse actors operating at different scales get involved in the crisis management process, making it multi-level. Hybrid forms of coordination and contestations emerge in multi-level governance in crisis responses that is contingent upon policy and political legacies (Liu et al., 2021). Thirdly, the actors are mainly concerned to bring “order” and a sense of “normality” rather than to ensure compliance with formalized rules (Gadinger, 2021) in such situations. Governance structures may aim to restore the pre-crisis status quo (based on reinstating “order and control”) or maintain the system or fix the experienced problems with patchwork like reforms at the margins of the legislative and policy structures (Bourbeau, 2018: 29).

This paper considers the 2015–2016 refugee emergency as a *governance crisis*. We acknowledge that the extraordinary flows of that period constituted an emergency that soon proved to be hard to manage with the existing governance capacity and migration policies. The inadequacy of the existing institutions and processes for dealing with the emergency paved the way to a governance crisis. We investigate this governance crisis as a process that opened up the floor for policy change through the redefinition of institutional roles, the transformation of pre-existing rules and norms as well as the emergence of new discursive frames. Thus, we zoom in on actors, legislative and policy structures and narratives to analyze how this crisis shaped migration governance in ways that have a lasting effect beyond the immediate crisis period.

The coupling of migration and crisis is not a recent phenomenon. The genealogy of the migration-crisis nexus shows that global migration was perceived as one of the ‘new’ threats challenging the international order and, thus, framed mostly as a security issue. This security lens formed the conditions of seeing and understanding the global migratory movements as a ‘crisis-generating phenomenon’ in the 1990s (Weiner, 1995). In the early 2010s, migration as a crisis became dominant in the media, policy and academic discourses to discuss migration in Europe (Cantat et al., 2020). At the global policymaking level, such a view of migration as a crisis, juxtaposed to migration as orderly and regular, is corroborated in the vocabulary adopted by the Global Compact on Safe, Orderly and Regular Migration (2018) and has been criticized for its rigidity and lack of touch with reality (Triandafyllidou, 2022).

As in the 1990s and early 2010s, the depiction of 2015–2016 as a ‘crisis’—regardless of being a fact to the extent that millions of people moved from their homes to seek protection and better livelihoods in other countries—has become an omnipresent lens in the public discourse for understanding migratory movements (Hagelund, 2020; Krzyżanowski et al., 2018). This particular 2015 ‘crisis’ gave stronger political impetus to the EU destination and transit states’ migration and asylum agenda. It opened a vast space for novel techniques of fragmentation, politics of categorizing, collective securitization and the launching of restrictive policies towards both migrants and asylum-seekers, often obfuscating the distinction between the two while failing to acknowledge the realities of mixed migration (Crawley & Skleparis, 2018).

While the above elements of securitization (Cantat et al., 2020), an excessive emphasis on migration control (Geddes & Hadj-Abdou, 2018; Paul & Roos, 2019), and border deaths (Pécoud, 2020) have been there for the last 20 years. We argue that the ‘governance crisis’ leads to ‘crisis’ as a mode of governance which intensifies these features of securitization and deterrence by normalizing exceptional defensive instruments such as push-backs, detention, accelerated asylum procedures, not only at the EU’s external borders but also leading to criminalization and dehumanization also within the EU (Pallister-Wilkins, 2015; Triandafyllidou & Dimitriadi, 2014). As Bello (2020) underlines, the securitization of migration is spiralling, involving an array of actors, discourses, policies, and practices embedded in a prejudiced narrative of migration in the times of crisis.

This paper seeks to take scholarly inquiry into the ‘crisis’ governance of migration a step further by investigating what are the actual characteristics of a ‘crisis mode of governance’. Can we identify the specific features that form this crisis mode? This paper focuses on the admittedly over-researched migrant and refugee emergency of 2015–2016, adopting though a broader temporal and spatial lens. We look not only at European transit points (like Greece, Italy and Hungary, Poland) or major destinations (Austria, Germany, Sweden and to a lesser extent the UK) but also at destination countries in the region, notably Lebanon, Iraq and Turkey. In addition, we do not focus on the 2015–2016 period only but extend our focus to the four years before and after covering the whole period between 2011 and 2018. The reason is that the countries in the region had felt the pressure of the Syrian civil war already since the mid-2011 and had gone into ‘crisis mode’ for dealing with the massive population flows. Expanding the focus of our inquiry we avoid over emphasizing a specific turning point (like that of fall 2015–early 2016) where a particularly acute emergency became a game changer.

**Fig. 1:**
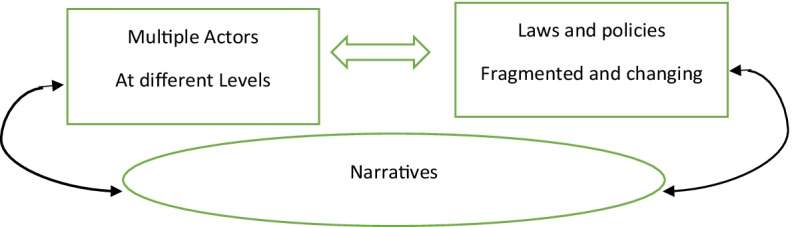
The ‘crisis’ mode of governance

Based on a meta-analysis of eleven country cases, we explore in what ways each country sought to manage the ‘crisis.’ The database used for this article is a compilation of the results from RESPOND project bringing together 78 country reports, 6 comparative thematic reports in the referred sub-fields of governance (border management and controls; reception, protection and reception) and several reports on specific sectors (e.g. housing, labour market, public health). For each sub-field and sector of migration governance, we examine three dimensions: actors, laws and policies, narratives. We investigate which were the actors involved in each country (public, private, hybrid organizations) and at what level (local, federal, national, regional and global) they operated; we look at policies on paper and practices on the ground and how they evolved as the flow of people grew during the years under study, in each country and seek for similarities and differences among these policies. We also focus on the discursive framing of asylum and migration issues in this period. We understand the connection between actors, laws/policies, and discourses as an interactive one (see Fig. 1).

Our attempt to develop a descriptive at least framework of ‘crisis’ as a mode of governance for migration arises from our critique of the Multi Level Governance approach (both type I and II) (Hooghe & Marks, 2001; Panizzon & Van Riemsdijk, 2018). Adopting MLG as our initial analytical framework in the RESPOND Project revealed several shortcomings. The first problem was that MLG treats governance from a static perspective, ignoring largely the temporal changes and complexities of interactions shaped by perceptions and power relations. Temporality is at the heart of the migration governance, particularly in a time of crisis (Sahin-Mencütek, 2018). MLG concentrates on current problems in public policymaking, assuming that the improvement of coordination and cooperation among actors is the goal and the ideal solution. Doing this, it fails to reflect on the conflicting and competing discursive frames that impact governance. It overlooks the interactive relationship between governance and narratives (see Fig. 1 above).

MLG also tends to simplify of the complex interactions among actors because it “sheds light on the possible patterns in vertical relations, while it does not effectively explore the horizontal relations, which are however crucial, especially at the local level” (Campomori & Ambrosini, 2020: 1). Third, The MLG approach partially overlooks the historical trajectory of migration and geopolitics that are crucial components of the governing of migration in regions encountering massive displacements from neighbouring countries such Turkey, Lebanon, Jordan or Iraq (Fakhoury, 2019). Moreover, due to MLG’s focus on levels and actors operating on the global, national and local scales, it fails to zoom into the micro practices or informal governing components (Fassin, 2011; Tazioli, 2019). Our development of a ‘crisis mode of governance’ seeks to overcome these shortcomings and to point to these complex dynamics as they emerged in the particular crisis period and to this day.

The paper starts by presenting the case studies and empirical materials on which our meta-analysis rests and the analysis method. The following sections discuss the migration governance features identified in the eleven cases analyzed, focusing on answering our three main research questions: who were the actors involved in the governance of the ‘crisis’ (we put the term in inverted commas to signal that it is a contested term); how did the legal provisions and policies evolved to deal with the emergency; and what were the narratives of governance promoted by the actors involved. In the concluding section, we identify the main features of what we call a ‘crisis modality’ for governing migration and asylum and discuss the implications of such a governance mode for the future of migration and asylum.

Before moving to the research design, a note on terminology is in order here. We speak interchangeably of migrants, asylum seekers, and refugees. We recognize as before 2015, the flows were mixed, and the motivations were also mixed (Van Hear, 2011) regardless of how they were framed by policy and media discourses put in specific, distinct socio-political and legal categories (as guests, displaced people, asylum seekers, refugees, labour migrants, irregular migrants) and in binaries (e.g. forced/voluntary; regular/irregular migration).

## Research design and methodology for meta-analysis

This article is based on a meta-analysis of data produced within the framework of the research project RESPOND, which included eleven countries along the so-called Eastern Mediterranean Route. The project developed country case studies in established EU member states, such as Germany, Austria, Sweden, Italy, Greece, and the United Kingdom (before Brexit), more recent member states, such as Hungary and Poland, and third countries, such as Turkey, Lebanon and Iraq, which have played a significant role as a source, transit and host countries of migrants and refugees. The countries are selected because we expect to find that they might have governed the massive arrival of migrants differently. The established EU member states have a common asylum regime and long-time destination for migrants and asylum seekers from the Eastern Mediterranean route. As new EU members, Hungary and Poland are still in the process of harmonizing their legislation with the EU scheme and do not yet have an established institutional asylum architecture. The topic of asylum and public debates on immigration had been of minor significance compared to established EU members. Regarding the expected crisis governing, the scenes in the non-EU countries like Turkey, Lebanon, Iraq are slightly different. Many of those arriving in Europe During 2015, mainly massive numbers of Syrians and Afghans (in the case of Turkey) had been already displaced to these countries since 2011, and the countries also act as both transit and origin country for migrants heading to Europe. They also have weak legal and institutional national asylum regimes, although having a host of protracted refugee situations for decades.

The database used for this article is a compilation of the results from RESPOND project (for further details, see Availability of data and materials at open access repository). The empirical data for country reports were obtained from legal and policy documents collection and analysis (desk research), in-depth interviews with 220 stakeholders, information exchange meetings with actors serving in migration governance, and 535 refugees. The project focused on three levels of governance: macro (legal and institutional frameworks and policies), meso (the implementation of policies by and everyday practices of state agencies and civil society actors) and micro (experiences of refugees) in each country regarding four policy fields (border management and control policies, reception, protection and integration. This article mainly reflects on the findings of macro and meso level; thus, it does not discuss findings from interviews with refugees for the sake of word limits in this article, making it impossible to present variation in the experiences.

Before discussing the main features of the modes of governing the ‘crisis’ across countries and policy areas, the search and coding process is briefly summarized to give a sense of meta-analysis (details in the Codebook, which is not public). Each country report of RESPOND Project has a section on migration and asylum policy developments. In this section, European country reports mapped out what were the main features of migration and asylum governance in the period before 2014, which policies were substantially changed with the crisis period 2015–2016 (such as bringing law proposals, practices about border controls, creating new categories, introducing new institutions or new cooperation modes, testing novel practices) and which continued to exist after that period and until 2018. For non-EU countries (Turkey, Lebanon and Iraq) 2011 was the starting point, 2015–2016 was a turning point because of the magnitude of the flows and reverberation in the region, and similarly to EU countries we look at policies that continued till 2018.

For all three areas (actors, laws and narratives), national research teams collected secondary data and conducted stakeholder interviews. We assessed changes over time on the basis of analyzing legal texts—including court decisions and parliamentary debates—and their interpretation by stakeholders. The analysis of policy texts and interviews with institutional actors gave also insights into the role of each actor and interactions with one another, at different scales (local, national, transnational). The discursive frames—the narratives of the crisis—were identified through qualitative discourse analysis of media materials, political speeches, and policy maker interviews.

These different types of materials, notably legal texts, media and interview transcripts were analyzed by using a common code structure. First level coding referred to the policy fields (border management and migration control, protection, reception, integration). The second level coding referred to the five indices: (1) main themes/narratives related to the policy field, (2) key actors involved and their roles, (3) cooperation between local, national, transnational as well as state and non-state actors, (4) problems during implementation, (5) suggested solution/best practices. These categories were selected to fully capture organizational, political and implementation dimensions of migration governance. Each category at the second level was also broken-down into sub-categories. For example, in the protection field, some themes include access to the asylum system, protection types, duration of procedures, detention and appeals. National research teams were given the flexibility to create their sub-codes or eliminate some, allowing room for inductive-deductive coding. Comparative reports built on the country reports focusing on similarities and differences across countries. They identified relevant commonalities, particularly changes in similar directions, such as restrictiveness in the legislation or policies, levels of decision-making and the actual implementations and practices. The comparative reports on reception, protection, and narratives also created typologies and country clusters to give a better understanding of patterns.

This article builds on these briefly described metadata of country reports (n = 78) and comparative thematic reports (n = 6). Using NVivo, a qualitative analysis software, we started our analysis with the country reports. First, we inductively coded the common section in each country report addressing the significant changes in migration policies between 2011–2018 and the reports’ executive summary and introduction sections. This allowed us to identify recurring themes such as legal fragmentation, policy gaps, multiple actors, externalization, outsourcing, privatization, protection challenges, barriers to access asylum, temporality, uncertainty, precarity, the crisis notion, the centralization, nationalization, localization, Europeanization, burden sharing, informality, securitization, criminalization, limitation on rights and others. Then we searched for these themes in the other sections of country reports, ensuring substantive content about these issues. Following that, we turned our attention to the comparative reports database for the same codes. This thematic mapping gave us insights into which themes persisted across country cases, policy fields and typologies.

The analysis is iterative. The discussions in the migration scholarship, specifically MLG and crisis governance literature inspired us to use such coding in interpreting the data in writing reports and to focus on them while we are thinking about parameters in meta-analysis. For this article, we categorized the themes under certain groups: actor architectures (e.g. private, public, international); the axes of legal/institutional structures (e.g. organisational-temporal); interactions (e.g. contestation, adaptation) in and between scales (e.g. local, national, transnational); asylum/reception profiles (Adhoc, regulative, restrictive) and narratives. The iterative inductive and deductive analysis indicated the persistence of three common patterns: changing actor landscapes, increased complexity and fragmentation in legislation and policy, and the return of a national control narrative as a critical element in governing the migration ‘crisis’.

The following sections discuss the different components of various countries' ‘crisis governance’ structures. We first focus on the actors involved; second on the changing laws and policies seeking common patterns in such reforms; third on the governance narratives that emerge in the countries under scrutiny.

## Multiple actors

The list of actors involved in the governance of migration in the EU and its eastern neighbourhood is extensive, including the EU institutions, intergovernmental organizations (IOs), governments, ministries, immigration and asylum offices, parliaments, parties, municipalities, courts (judges, lawyers, bar associations), humanitarian actors, rights-based groups, activists, refugee community organizations and others, forming a multi-level governance field (Gökalp Aras, 2020). Indeed, migration governance is typically a multi-level governance field which is characterized by interdependency and interaction between levels and actors (Caponio & Jones-Correra, 2018: 1996; Panizzon & Van Riemsdijk, 2018: 3). The question that arises here is how these actors relate to one another to address an emergency.

The country cases analyzed illustrate the involvement of actors from the EU and overall international level down to the federal/regional level to the province/city and the municipal levels into the migration governance. Four EU institutions, the European Commission, the Council of Ministers, the European Council and the European Parliament took substantial role along with the Member States. From 2011 to 2017, these institutions published at least 95 documents in the format of policy documents, proposals, speeches, ordinary conclusions, resolutions, directives, agreements, reports on immigration and asylum (Comparative Report from the RESPOND Project, hereafter Pannia et al., 2018: 85). The bone of contention in interactions is that traditionally, states are the main sovereign actors with exclusive legislative power in migration, asylum rights, and foreigners’ legal status. Overall, states decide on the rules of entry, exit, access to rights and the legal categories. Particularly in mass migration situations, states determine border rules or enforcement measures aimed at halting migration or asylum flows (Pannia et al., 2018). States also hold power to categorize people on the move as regular, irregular, refugee or temporary protection holders (Gökalp et al., 2020). The acquisition of permanent residency and citizenship is also a field under the jurisdiction of state agencies (Barthoma et al., 2020). Nation-states do not abandon their sovereignty claims over migration; hence there is a high level of centralization and renationalization in-country examples Austria, Italy, Turkey and Lebanon.

The centralization and renationalization of migration governance have nuances because it is selective regarding the policy sectors on the one axis, exercising legislative and executive power on the other axis. Besides the EU and central state authorities, sub-national entities (federal states, regions or municipalities) are involved in migration governance, specifically for newcomers’ reception and integration, but not necessarily for protection and border controls. Federal states like Austria, Germany, and Italy take more decisive roles in implementation but do not hold legislative power reserved for the central government. The exception was in a few cases such as Scotland, Wales and Northern Ireland who have the power to decide on housing, health care, education, children’s services and the social welfare of refugees and immigrants that might collide with the national legislation of the UK (RESPOND project Country Report (hereafter CR), Hirst and Atto, 2018). In Austria, access to civic integration is for local authorities to decide on objecting to the federal approach (Josipovic and Reeger, 2018). However, as in the case of Sweden, “there is a tendency towards the centralization of authority in areas previously allocated to local authorities” (Borevi and Shakra, 2019: 42). In Italy, despite the 2001 constitutional reform stressing the central government’s exclusive competence in migration affairs, regions play a decisive role in passing legislation on healthcare, education, children’s services and social welfare with the support of civil society (Ibrido and Terlizzi, 2019).

In all countries, non-state actors (civil society movements, migrant/community organizations, bar associations and a large body of volunteering organizations) not only monitored and criticized legal disparities and arbitrary practices in migration governance but also filled in the gaps, especially in the service sector (health, education, aid, first response etc.) where governments failed (or lacked) to provide these services (Gökalp et al., 2020). Despite the non-state actors and IOs intensive supportive role in the reception of refugees in the initial phases, such as in Greece, Germany and Italy in 2015–2016 and in Turkey, Lebanon, Iraq in the 2012–2015 period, their motivation and resources faded away over time. Also, the central states gradually provide control over fields such as refugee education and social assistance that non-state actors in the early phases governed. Striking examples include the Lebanon government ordering the UNHCR to stop registering in 2015 and Turkey closing down all education centres run by non-state actors in 2015–2016 (Rahme, 2020; Rottman, 2020).

We noted the outsourcing and partial privatization of migration governance-related services, such as health, catering, accommodation or security, in several of eleven country cases (Chemin and Nagel, 2020). In the UK, three private companies manage the entire asylum reception system (Foley, 2019). In Germany, accommodation centres and asylum consultations benefit from the services of private companies (Hänsel et al., 2019a, 2019b: 41). Outsourcing immigration-related services to the private sector create a mixed web of contractors and subcontractors with (limited) coordination with central asylum authorities and regional and local municipalities (Hirst and Atto, 2018). The international IOs and NGOs also outsource their services to the private sector when the need arises.

From a governance perspective, top-down and bottom-up initiatives are present simultaneously. However, they show differences based on sub-issues areas, making the scene more complex, messy and hybrid (Scholten, 2020). The increased involvement of non-governmental actors in crisis governance is common across all cases, but their potential to resist power hierarchies or create counter-narratives depends on their resources and the socio-political context on the one hand, sector on the other. (Gökalp Aras, 2020; Sahin Mencutek, 2021). While relations between state and non-governmental actors tend to be smoother in sectors like reception and integration in all counties, the border control and protection sector seems more concerning for actor relations, particularly for frontline countries, as observed in criminalizing of humanitarian actors in Italy or stopping the UNHCR’s registration activities in Lebanon. A contestation between the state and (excluded) non-state actors are more apparent when the latter monitors the former’s practices from a human rights point of view. In the field of reception and integration, states invite new non-state actors, such as refugee community organizations, into the process on a case-by-case basis for carrying out subsidiary role, returning to the normal state of affairs once the crisis is over by restricting their activities.

The eleven country cases pointed out the need for well-structured coordination and clear responsibility-sharing between various actors to remedy better migration governance if they are coupled with transparency and the right-based approach. As indicated in other recent studies (Garcés-Mascareñas & Gebhardt, 2020; Oliver et al., 2020), our country cases show that reception and integration policy fields can be specifically benefited for multilevel governance, mainly when local engagements and networks serve as “policy entrepreneurs”. Given that adequate coordination mechanisms are often lacking, this multiplicity of actors undermines the uniformity of practices and often results in substandard services and uncertain rights. Almost in all contexts, the relations are mostly path-dependent. As discussed in migration scholarship, multi-level governance faces trouble, turning some policy fields such as reception into a “battleground” for involving actors (Campomori & Ambrosini, 2020).

Our analysis of the eleven country cases suggests the following dominant features regarding actors’ involvement and crisis governance in migration: (1) Nation-states remain at the centre of policymaking processes. (2) Transnational actors (e.g. the EU) play an intermediary role—which can turn into a role of meta-governor, setting the (new) norms and mechanisms for migration governance in an increasingly interdependent world. (3) There is increasing resistance at the local level against top-down migration policies leading to both fragmentation and policy innovation in governance structures. (4) Non-governmental actors are involved in this process at least in two ways (a) in a neo-liberal governmentality mode, they contribute to the development of a control regime or (b) by resisting and exercising an external governance mode by monitoring humanitarian values and raising a counter-narrative against restrictive policies or anti-immigrant discourses. Migrants are also involved in resistance through everyday struggles, coping mechanisms and solidarities challenging border and asylum practices.

A multilevel governance perspective enables us to identify actors and issues related to coordination. However, it fails to fully capture the complexity of policymaking and interactions among levels (Scholten, 2020). Importantly, it rarely links the governance with migration politics that show us the relevance of public attitudes, narratives and competing interests for managing migration. Therefore, after reviewing the legal and policy provisions in the next section, we shall turn to a closer inquiry of the governance narratives and how they contradicted the complex set of actors involved and the fragmented legal provisions.

## Complicated and fragmented laws and policies

Both international and national legislations are key to migration governance. Except Iraq and Lebanon, many countries in the sample are signatories of the 1951 Geneva Convention and its additional protocols. Turkey is an outliner due to be retained a geographic limitation to its ratification, meaning that it grants refugee status only to those fleeing from European countries and provides conditional refugee status until they will be resettled in third-safe countries. Many countries (except Iraq and Lebanon) recognize the European Convention of Human Rights (ECHR), together with its principle of protection against torture or inhuman or degrading treatment or punishment, as essential safeguards. All EU countries are bound by the EU acquis that includes the Common European Asylum System (CEAS) that establishes common minimum standards for asylum and the operation of the Dublin Regulation As a non-EU country, the UK is only part of the first phase of the CEAS comprising the Refugee Qualification Directive (Directive 2004/83/EC), the Asylum Procedure Directive (Directive 2005/85/EC), and the Asylum Reception Conditions Directive (Directive 2003/9/EC).

Despite a level of policy convergence regarding legislation emanating from international and EU frameworks, the comparison of national asylum regimes of the countries shows evidence of potentially contradicting modes that can partially explain by deviation from supra-national legal frameworks during their adoption at national levels. The differences also emerged as the result of multiple, fragmentary normative stratifications, jeopardizing internal consistency and effectiveness, while few of them improve the rights of asylum seekers (Josipovic et al., 2018). Unlike what one might expect, the changes in legislation were not necessarily coherent or clear. Moreover, for both EU and non-EU countries, national legislations have been frequently updated, amended, changed and revoked.

The impact of complex legislation is mainly felt in asylum policies. Governments extended their adoption of diverging labels for migrants as protection seekers, guests, displaced persons, economic migrants or illegal migrants. These made the nexus between irregular migration/asylum/mixed migration more ambiguous than before (Gökalp et al., 2020). The legal changes complicated bureaucratic procedures and extended the duration of decisions as countries aimed to reduce asylum applications. Additional accelerated, fast-track and border procedures were introduced to prevent and restrain access to international protection and speed up asylum applications and assessments (ibid.). In many countries, permanent protection schemes have been replaced by subsidiary and temporary protection mechanisms. The Qualification Directive introduced subsidiary protection in 2004, and it sets out protection for certain individuals who do not satisfy the 1951 Convention refugee definition, rather than bringing such persons into an elaborated refugee definition. The rights connected to subsidiary protection status are much more limited than those coming with asylum or refugee status. Our research showed that Germany, Austria, Sweden, Poland, Greece, Italy and the UK widely used this type of protection (Ibid., pp. 31–32). Confirming the findings of previous research (Kreichauf, 2018), the temporary nature of the protection provided in these countries has led to practices of containment being the norm, living standards being very poor, and asylum seekers being marginalized.

Temporary protection forms have become the primary instrument for governing migration in crisis not only in the EU but also in non-EU countries. Temporality is normalized and mainstreamed as the main mode of governing the flows of asylum seekers (Gökalp et al., 2020, p. 35). Turkey governs 3.6 million Syrian refugees through its national temporary protection regulation, while Lebanon has treated almost one million Syrians with a de facto temporary protection regime since 2014. In 2016, Sweden introduced the Act called Temporarily Restricting the Possibility to Obtain Residence Permits in Sweden, which aims to reduce asylum seekers by only issuing temporary residence permits and restricting family reunification (Favilli, 2018).

Almost in all countries, the legislative structures rarely reflect constructive, participatory law-making processes because parliaments, civil society, and refugees have a limited say on policy changes, such as in the UK, Sweden, and Greece. Parliamentary scrutiny or debate has also been circumvented in other ways: Recent regulations have been mainly developed via secondary legislation (e.g., bylaws, decrees, circulars, regulations, guidelines) as in Turkey or Italy. In almost all countries, legislation is decided on by governments and implemented often by the ministries of the interior that are well-known for having security-oriented lenses in approaching migration issues. Secondary legislation is rarely subject to parliamentary debate. Both decision-making and implementation are all concentrated in the hands of the executive, sometimes facing challenges from national or international judiciaries.

Since 2015, contested ad hoc external cooperation instruments, such as statements, deals, compacts, joint actions, joint declarations, have also intensified, such as the EU-Turkey Statement of 2016, Joint Action Plan agreed between Turkey and EU, join return operations of Frontex and Greek authorities (Pannia et al., 2018). These arrangements fall outside the ambit of international refugee law and the EU Treaties for migration governance. They are often designed in a way that not only contradicts the EU norms and standards but also sidelines the European Parliament or the European Court of Justice (CJEU) (Ibid.). This is quite a unique feature noted in this crisis whereby informal arrangements are adopted as a policy innovation to address the crisis of migration governance.

We should note that countries we studied show both compliance and non-compliance towards the meta-governor mission of the EU. The degree of compliance is higher in the established EU members, while contestation, partial compliance or decoupling are not rare for new member states like Hungary or Poland. The non-EU countries, including the UK, illustrate substantially different policy environments across all sectors and few signs for being receptive to the EU’s meta governor role. For Turkey, Lebanon and Iraq, the financial incentives or conditionalities make a difference in adopting norms and mechanisms of the EU, but they are far from being a passive receivers of EU policies (Gökalp Aras, 2021). These pattern confirms what scholars of European integration and integration have long debated: there are internal differentiation within the EU as the exceptions that member states carve out from EU law through opt-in and opt-out possibilities, while external differentiation refers to a situation in which third countries import some European rules accompanied by compensation payments (Leuffen et al., 2013). The dynamics of differentiations within and beyond Europe are predominantly sector-specific and functionalist (Lavenex, 2015: 836).

In summary, the legal framework concerning migration and asylum/international protection in all the countries under study became extremely complex and hypertrophic. Legislation has been changing continuously and often incoherently, frequently lawmakers resorting to decrees instead of proper statutes/acts of Parliament. The result has been a fragmented legal framework, that cannot be consistently interpreted and implemented. The legal enforcement and guarantee of fundamental rights are jeopardized, eventually coming down to the discretionary power of single offices and individuals.

Bringing the two together, the diversity of actors stated above and the fragmentation of legal provisions create an uneven playing field where the national level emerges as dominant, despite an otherwise complex multilevel governance system. The following section analyses the related governance narratives as they have appeared in our country cases.

## Migration governance narratives: the return of the nation-state?

Not only legislative content and institutional actors but also narratives, framing and politics influence migration governance. Without focusing on discourses and politics behind governance, the analysis remains descriptive and partial. To overcome this, we take a discursive approach in this section before identifying governance tools in the issue area. There is no doubt that migration has been one of the most politicized European and domestic debate topics in each country under investigation. The core of the debate is that the increasing migration is approached as a crisis and something beyond member states’ capacities to respond independently. The crisis perception has emphasized migration narratives that dominate political debates, traditional print media, and social networking sites. Narratives may impact drawing boundaries, shaping public opinion, legitimizing exclusionary policies, and providing a better understanding of migrants’ experiences and identities. They are deeply embedded in knowledge production, policymaking, politics and power and shaped by them (Sahin-Mencütek, 2020).

Our study here relied on a qualitative analysis of political claims in seven EU member states involving various political parties in both government and opposition (Josipovic et al., 2022). The data analyzed consisted of public speeches accessed in available media sources (newspapers, press releases, websites). The speeches were examined to identify “how (1) politicians publicly refer to the EU’s institutional architecture as well as interstate relationships; (2) how they evaluate existing European and national policies and proposed new ones, and; (3) how they referred to immigrants and the domestic public as the primary audience of their speeches (ibid:67). The findings are quite nuanced. In the wake and aftermath of the crisis, migration policy narratives shifted towards more nationalist agendas despite the calls for Europeanisation, solidarity and burden/responsibility sharing. As it is not the first time, this is called as “renationalization” of migration governance. Renationalization advocates restrictive, illiberal immigration policies and securitization in Europe and elsewhere. Regarding policy impact, renationalization in EU countries triggered the decoupling modes in migration and asylum policies.

Renationalization is closely linked to the context of the rise of populism (Guiraudon & Joppke, 2001), in which migration is constructed as the main scapegoat for unemployment and societal failures (Cochrane & Nevitte, 2014). The securitization of immigration emerged as both an outcome and a cause of extremist narratives inside right-wing political parties (Estevens, 2018). What populist political elites and leadership models propose is more conservative governance models focused on modifying the role of the nation-state in the governance of migration to “fix the migration problem”. Particularly in the well-established destination countries (Germany, Sweden, Austria), this is intersected with discussions around social welfare, while austerity policy measures are more linked in countries encountering economic crises (Greece, Italy). In non-EU countries (Turkey, Lebanon, Iraq), the politicization of migration overlaps with debates and crises concerning geopolitics, domestic power-sharing and resources distribution (Rahme, 2020; Warda et al., 2019, Gökalp Aras, 2021).

The UK is an illustrative case for tracing concrete consequences of populist political discourses favouring the renationalization of migration governance (Favilli, 2018). The UK had previously opted out from the Schengen Agreement, the Economic and Monetary Union and the Area of Freedom, Security and Justice. Before the significant opt-out with Brexit (2020), the UK only abided by the first phase of the CEAS (The Refugee Qualification and the Asylum Directive) but opted out of the ‘Asylum Recast Package’ too (Gökalp et al., 2020). Nevertheless, post-2011 developments and the 2015–2016 crisis climate made the UK more reluctant to provide refugee protection (Rahme, 2020). Immigration and asylum debates appear as the most vital issues driving Brits to vote to leave the EU in 2016 and finally abscond on 31 December 2020.

Besides populism and concerns about the distribution of social welfare (to whom and how) in Europe, issues around identity/community and self-perception may directly or indirectly influence migration governance. They provide insights for understanding the social and normative logic of governance communities beyond the functionalist mode of governance. National, regional or local authorities do not adapt their policies only according to a functional need rather they are influenced by broader narratives on identity, citizenship, and politics. Specifically, in the front states such as Poland and Hungary, which have faced migration pressure, migrants (mainly Muslims) are constructed as “complete outsiders” which has created tension since 2015–2016. These countries do not want to self-identify as buffer zones or waiting rooms for migrants attempting to reach Western Europe.

The renationalization of migration governance is closely linked to conflicting discourses around Europeanisation. The comparative analysis on Europeanization illustrates that government actors have developed both liberal and conservative Europeanisation discourses to stay in line with the humanitarian role of the EU based on the solidarity principle and to strongly prioritize a security-focused national agenda (Josipovic et al., 2022). The discourses are not sole political claims or normative positions. Instead, they serve as the basis of justification and legitimization of essential policy decisions seen in Brexit in the UK. Although there are differences regarding the level of migration and asylum governance, particularly the shortcomings of the EU in dealing with the crisis (in particular the failure of the Dublin system, the hotspots approach, etc.), mainstream public opinion in the majority of the countries remains pro-European. However, domestic politics (e.g., Poland, Hungary, Greece) is detrimental to the multilevel governance of migration, which has strengthened the inter-governmentalist discourses among the EU Member States (Josipovic et al., 2022).

In the non-EU countries, the content of the renationalization of migration governance via a restrictive turn has shown some similarities. However, the driving forces are slightly different from those in Europe. First of all, these countries (Turkey, Lebanon and Iraq) had faced massive displacement since 2011–2012, earlier than European states in 2015–2016. However, they pragmatically underlined humanitarian concerns and did not call arrivals a ‘crisis’ until the latter group put the terminology into broader circulation (Korkut, 2016). Security risks and economic fragility in the former group concerning actual crisis-engendered displacement (Syrian war) have some factual basis because they share long borders and socio-economic relations with Syria. The refugee issue is highly embedded in these countries' geopolitical context, depleting national capabilities and domestic power dynamics, making migration governance more sensitive, conflictual and temporal (Sahin Mencutek, 2018). The regional destabilization and deterioration of insecurity at borders over time and the subsequent influx resulted in stricter entry rules and harsh legal residency requirements after 2014–2015, despite ethnic, kinship, clan, and religious ties between host and refugee communities.

Additionally, the closure of trade routes between Syria and Lebanon has led to a sizable pressure on the Lebanese economy that has intensified the already existent political polarisation and gridlock (Rahme, 2020). In Iraq, the response to the influx of Syrian refugees was fragmented and motivated by ethnic or local political agendas, as demonstrated by the Kurdish Regional Government’s treatment of Syrian Kurd refugees. Also, internal displacement inside Iraq exceeded five million persons after the ISIS violence required additional measures (Warda et al., 2019). Turkey’s geopolitical-security anxieties about possible Kurdish self-administration in Northern Syria, close to the Turkish border and failure to garner support for building a ‘safe zone’ played a role in responding to Syrian migration (Sahin-Mencutek, 2018). In the end, domestic and regional concerns gave reasons for these countries to re-nationalise their migration governance along with restrictive policies similar to those of their European counterparts. Analyzing the governance narratives as we have done above reveals an essential contradiction or tension between, on the one hand, fragmented legal systems and a multiplicity of actors and, on the other hand, a re-assertion of the sovereignty and power of the nation-state.

## Concluding remarks

We argue in this paper that the ‘crisis governance’ of migration is not simply a narrative or a representation that guides policy choices but is emerging as a mode of governance with specific characteristics. Through the meta-analysis of a broad set of materials arising out of the RESPOND research project, we identified three salient (migration) governance features in times of crisis. These are (1) a complex actor landscape bringing together state, civil society, and the private sector, at national, local and international levels (2) complicated and fragmented legal systems and policy provisions that may vary both at the temporal level (as they change frequently) but also at the territorial level (different provisions are in place in different areas within a given country); (3) a renationalization narrative that seeks to bring this multifaceted and fragmented governance landscape together under the promise that the national state can re-establish control and solve the ‘crisis’. These three features of the ‘crisis’ mode of governance interact with one another. Interestingly while the first two suggest a certain level of tension and confusion, the third element, the narratives, seeks to re-establish order and control rather than acknowledging the messy nature of the problem, the uncertainty that prevails and the difficulty or impossibility to control the flows effectively.

However, this is not a contradiction but rather an inherent feature of the ‘crisis’ mode of governance: it hides the messiness and uncertainty and presents an ‘order and control’ façade that disguises the ‘crisis’. Although our study shows that the governance of people on the move, whether asylum seekers, refugees or migrants, necessitates the delegation of state’s responsibilities to non-state, private or intergovernmental organizations, in different policy domains, this does not necessarily mean the discursive withdrawal of the nation-state or the elimination of all other non-state actors generating counter-narratives. Instead, the narrative seeks to re-establish the order at the discursive level hiding the messiness at the policy implementation level on the ground.

The three features of the ‘crisis’ mode of governance in migration and asylum point to two inherent elements in it: uncertainty and temporality. The ‘crisis’ mode involves ambiguous legislation, excessive discretionary power at the implementation level, constantly changing provisions, and delegating to civil society and the private sector with overlapping or contradictory functions. The ‘crisis’ mode in governing migration allows nation-states to privilege ad hoc measures, without a clear plan or without assessing the impact of such policies on the migrants or refugees, stretching the limits of existing rights-based legislation, privileging ‘informal’ (restrictive) policy modes instead. At the same time, in many country examples such as Turkey, Greece, Lebanon in our study, we have observed how temporariness becomes a ‘permanent’ situation and how instances of Sweden, Austria, Germany look for gradually replacing their permanent protection regime with the temporal one. This temporariness opens wide room of manoeuvre to the migration governors—not only states, IOs but also NGOs taking up the role of intermediaries—to test novel policies and practices for controlling the entry of people on the national territory, regulating their stay and maintaining order. Solidarity groups and migrants have to navigate temporariness to cope with the situation and push for alternative understandings and counter-narratives. One overarching pattern is prevalent in almost all countries in our sampling, the ‘silent’ consensus of policymakers towards a more restrictive approach to asylum and migration policies favouring control and deterrence over humanitarian concerns and rights. This combines with the weakening of global and supranational governance structures (e.g., intergovernmental instead of a community approach in EU governance). At the same time, it is the very crisis mode that violates legal and institutional norms and processes and even at times constitutional principles and international conventions (the EU-Turkey statement being an illustrative example of such modalities). In the end, the ‘crisis’ mode turns into an ‘inevitable’ reality, uncertainty and temporality become the ‘new ‘normal,’ and also eventually the governing norm for migration.

Identifying the main features and inherent contradictions of the crisis mode of migration governance is particularly relevant and timely today as we seemed to go through a chain of crises started with the 2008–2009 international financial crisis, continued with the ‘refugee crisis’ of 2015–2016 and culminating with the pandemic crisis of 2020–2021. Interestingly the pandemic crisis is also a migration crisis: Some migrants have remained stuck at origin or destination or were forcefully repatriated. Asylum seekers were detained in camps for fear of contagion, often with inadequate health services and restricted access to vaccines. Other migrants were brought in through chartered flights to fill in the jobs that were ‘essential’ for the food chain or the care provision in the main destination countries (Triandafyllidou, 2022). The ways in which the pandemic crisis is being governed seems to follow the main features of the crisis mode analyzed in this paper: multiplicity of actors, fragmentation of legislation, and renationalization restore ‘order’ ‘normality’ and sovereignty. Understanding better the 2015–2016 refugee emergency and how it has led to the full-fledged development of a crisis mode for governing migration can provide insights for future research on the crisis governance of migration under the pandemic.

## Data Availability

The datasets generated and/or analysed during the study. The datasets generated and/or analysed during the current study are available in the [ZENODO] repository, https://zenodo.org/communities/respond/?page=1&size=20]. It is also an open Access in the DiVA repository of Uppsala University, https://uu.diva-portal.org/smash/search.jsf?dswid=5077.
